# Experimental Characterization, Modeling, and Numerical Evaluation of a Novel Friction Damper for the Seismic Upgrade of Existing Buildings

**DOI:** 10.3390/ma16051933

**Published:** 2023-02-26

**Authors:** Eleonora Bruschi, Luca Zoccolini, Sara Cattaneo, Virginio Quaglini

**Affiliations:** Department of Architecture, Built Environment and Construction Engineering, Politecnico di Milano, Piazza Leonardo da Vinci 32, 20133 Milan, Italy

**Keywords:** friction damper, lead damper, energy dissipation, reinforced concrete, steel frame, seismic upgrade, non-linear analyses, OpenSees

## Abstract

The paper presents the experimental characterization, the formulation of a numerical model, and the evaluation, by means of non-linear analyses, of a new friction damper conceived for the seismic upgrade of existing building frames. The damper dissipates seismic energy through the friction force triggered between a steel shaft and a lead core prestressed within a rigid steel chamber. The friction force is adjusted by controlling the prestress of the core, allowing the achievement of high forces with small dimensions, and reducing the architectural invasiveness of the device. The damper has no mechanical parts subjected to cyclic strain above their yield limit, thereby avoiding any risk of low-cycle fatigue. The constitutive behavior of the damper was assessed experimentally, demonstrating a rectangular hysteresis loop with an equivalent damping ratio of more than 55%, a stable behavior over repeated cycles, and a low dependency of the axial force on the rate of displacement. A numerical model of the damper was formulated in the OpenSees software by means of a rheological model comprising an in-parallel system of a non-linear spring element and a Maxwell element, and the model was calibrated on the experimental data. To assess the viability of the damper for the seismic rehabilitation of buildings, a numerical investigation was conducted by performing non-linear dynamic analyses on two case-study structures. The results highlight the benefits of the PS-LED in dissipating the largest part of seismic energy, limiting the lateral deformation of the frames, and controlling the increase in structural accelerations and internal forces at the same time.

## 1. Introduction

The implementation of energy dissipation devices inside structural frames has proven to be an effective technique for improving the seismic behavior of existing constructions [1,2,3,4,5,6,7,8,9,10]. Generally speaking, energy dissipation devices can be categorized into two families, namely fluid viscous dampers, which response depends on the rate of deformation, and hysteretic dampers, which response mainly depends on the magnitude of deformation [11]. While fluid viscous dampers are more popular for strategic structures (such as hospitals, police stations, and fire stations) and high-rise buildings [12,13,14,15,16,17,18,19,20,21], hysteretic dampers are generally used for the retrofit of ordinary buildings, as demonstrated in numerous studies [22,23,24,25,26]. In framed structures, energy dissipation devices are usually incorporated within steel braces installed between consecutive floors [11], which permits the activation of the dampers by exploiting the relative displacement between the stories. The combination of steel braces and energy dissipation devices is beneficial since the braces provide an increase in structural stiffness that reduces structural displacement, while the dampers, through the dissipation of seismic energy, dampen vibrations and reduce structural acceleration [10,27,28,29]. However, the installation of damped braces in existing structures requires an important amount of construction work, resulting in significant disturbances for occupants and critical alterations to the building layout because of their excessive dimensions, which ruin the esthetic and architecture of buildings [30].

Another important drawback of damped braces is the increase in internal forces in the structural elements that surround these dissipative systems, which often need local strengthening, especially at connections that are particularly sensitive to stress concentration [2,7,31,32]. Moreover, the stiffening effect of these braces decreases the vibration period of structures, causing an increase in horizontal forces at the foundation level [30]. This requires further expensive interventions to strengthen the foundations of the main frame [7,30,31,32,33].

Among the various types of current hysteretic dampers [24,29,30,31,32,33,34,35,36], buckling-restrained braces (BRBs) are perhaps the most popular system and are used for both new and retrofitted structures [2,37,38,39]. BRBs dissipate seismic energy through the inelastic deformation of a mild steel core confined in a rigid metal sleeve, which provides buckling resistance and allows the development of large and stable hysteretic loops with an almost symmetric hysteretic behavior [37,38,40,41,42,43], providing an equivalent damping ratio on the order of 20% to 40%. Due to their widespread use, modern codes for seismic retrofit (e.g., ASCE 41-17 [44]) and seismic design (e.g., AISC 341-16 [45], ASCE 7–16 [46]) have incorporated general guidelines for the design of BRB frames. However, BRBs are susceptible to low-cycle fatigue failure due to their limited cumulative ductility capacity [47,48,49,50,51,52], which limits their survivability in case of long-duration or repeated seismic sequences, possibly leaving a structure unprotected in the case of, e.g., main shock–aftershock sequences.

Some authors of this paper have recently proposed a novel damper, named the PreStressed-Lead Damper (PS-LED), which dissipates seismic energy through the friction force triggered between a moving shaft and a lead core [3,4,5]. Because of an essentially rectangular hysteretic cycle, the damper is expected to provide an equivalent damping ratio close to 60%. Moreover, since there are no mechanical parts subjected to inelastic deformation, this device is insensitive to low-cycle fatigue, and, therefore, it can survive multiple design earthquakes.

In the present study, the PS-LED was characterized following the recommendations of the European standard EN 15129 [53], which is compulsory in Europe for the CE Marking of anti-seismic devices, and its viability for the seismic retrofit of existing structures was investigated via numerical analyses. In particular, Section 2 reports the results of an experimental campaign on a prototype of the PS-LED and the formulation of a numerical model aimed at representing the constitutive behavior of the device in the OpenSees software program [54,55]. In Section 3, two structures, including a reinforced concrete (RC) residential building and a steel moment-resisting framed (MRF) office building, are taken as the case studies and retrofitted by means of Chevron braces equipped with the PS-LED. The seismic upgrade of the two buildings is performed at the ultimate limit state in compliance with the Italian Building Code (NTC-18) [56], and the effectiveness of the damper is assessed by means of non-linear analyses.

## 2. Characterization and Modeling of the PS-LED

### 2.1. Description of the Device

**Figure 1:**
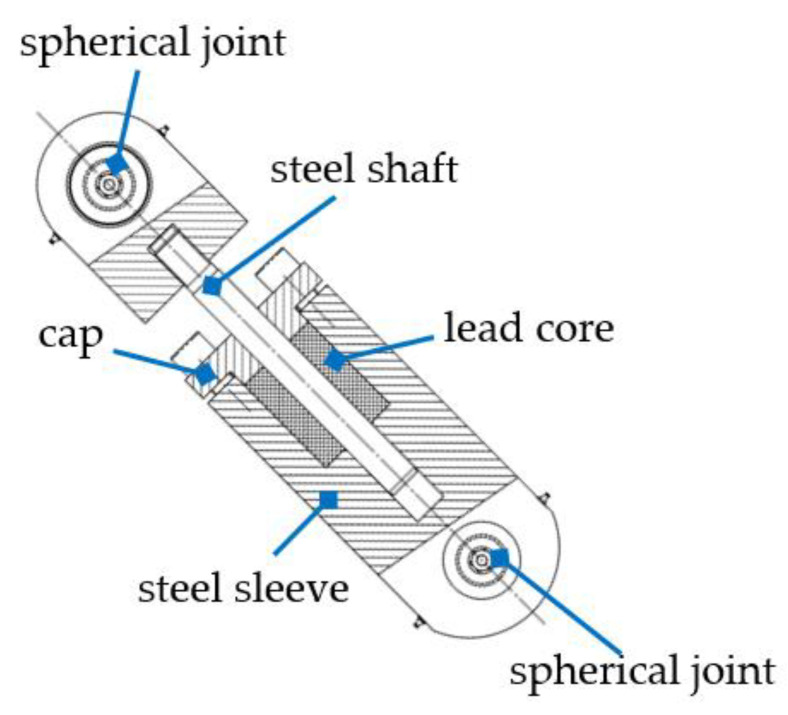
Sketch and nomenclature of the main elements of the PS-LED.

The PS-LED consists of a steel sleeve filled with lead, in which a steel shaft slides due to the effect of an external action, as shown in Figure 1. The linear motion of the shaft is driven by a bushing in the cap, which also prevents the leakage of lead. Spherical joints are provided at either end of the damper to prevent the transmission of bending moments.

**Figure 2:**
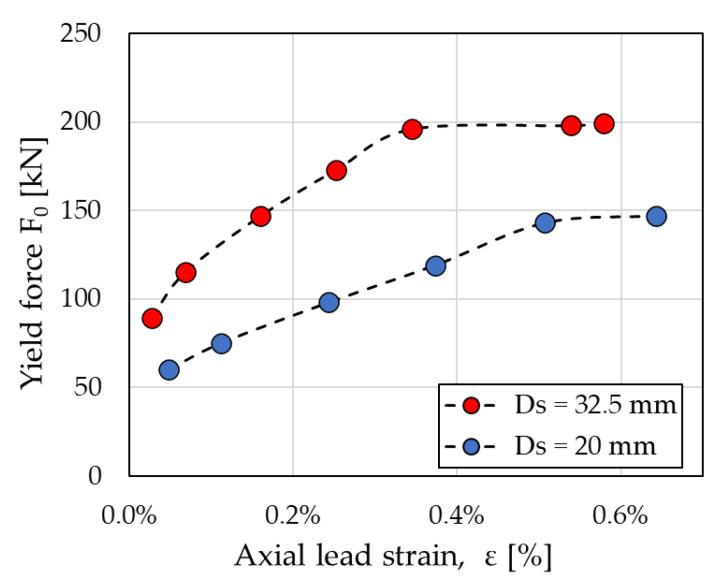
Effect of prestressing on the force of the PS-LED: yield force *F*_0_ vs. axial strain ε of the lead core calculated in the numerical analyses considering a shaft diameter (*Ds*) of either 20 mm or 32.5 mm (adapted from ref. [3]).

The lead core is prestressed after the assembling of the damper in order to remove voids formed during the casting process, resulting in a perfect fit to the sleeve and the shaft [1,3]. During the sliding of the shaft, the force *F*_0_ of the PS-LED is expressed by the formula of Equation (1):

(1) $$F_{0}=\mu_{k}\times p\times A$$

where *μ_k_* is the coefficient of friction at the interface between the shaft and the lead core; *p* is the contact stress at the interface; and *A* is the area of the lateral surface of the shaft sliding within the lead core. Prestressing the lead core increases the contact stress *p*, and hence the force of the damper, permitting the achievement of high strength combined with small dimensions of the device. Previous numerical simulations have demonstrated [3] that the force *F*_0_ of the PS-LED increases almost proportionally with the axial strain of the lead core ε until a certain strain level is reached, corresponding to the yielding of the lead core, and beyond which no further increase occurs, as shown in Figure 2.

### 2.2. Experimental Campaign

**Figure 3:**
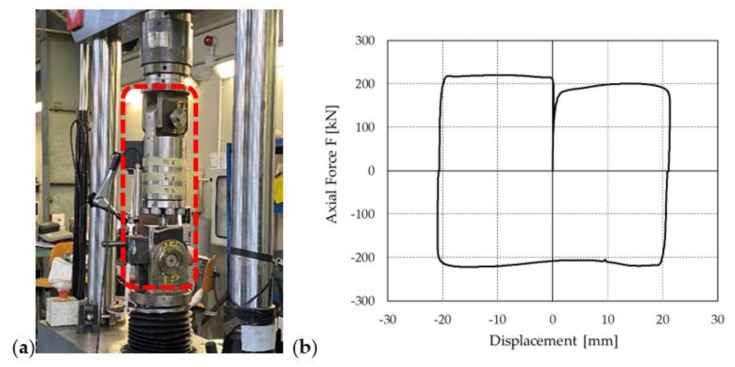
(**a**) The PS-LED prototype (highlighted with a dotted frame) on the testing machine, and (**b**) a typical force–displacement curve of the PS-LED.

A prototype of the PS-LED, which was rated for a nominal force of 220 kN and a seismic displacement *d_bd_* of 20 mm in either direction, was tested at the Materials Testing Laboratory of Politecnico di Milano, using a servohydraulic testing machine (MTS Systems, Eden Prairie, MN, USA) with a load capacity of 500 kN, as shown in Figure 3a.

The diameter of the shaft was *Ds* = 32 mm, the external diameter of the lead core was *D_L_* = 60 mm, and the length of the lead core was *L* = 80 mm. The materials were S355 carbon steel for the sleeve, 42CrMo4 steel for the shaft, and 99.99% pure lead for the core.

The typical experimental force–displacement curve of the PS-LED, as reported in Figure 3b, presents a linear initial branch, which corresponds to the elastic deformation of the shaft before sliding at the lead–shaft interface is triggered, followed by a zero-stiffness secondary branch corresponding to the sliding, with a constant friction, of the shaft within the core. The hysteresis loop is almost rectangular in shape, which maximizes energy dissipation for a given displacement amplitude. The damper force remains almost constant regardless of the accommodated displacement, indicating that the PS-LED is able to limit the stress increase in the structural frame in case the design displacement is exceeded during a strong earthquake.

The prototype of the PS-LED was subjected to the test protocol presented in Table 1. The Cyclic and the Ramp tests were performed in accordance with the provisions of the European standard EN 15129 [53]. The standard prescribes assessing damper properties by performing five sinusoidal cycles at both 25% and 50% of *d_bd_*, and ten cycles at *d_bd_* at the reference frequency of 0.5 Hz, corresponding to a period of a structural system in which the tested device has to be used for 2 s. Then, the prototype was subjected to a monotonic ramp with increasing deformation up to the amplified displacement *γ_b_ γ_x_ d_bd_*, when the amplification factor *γ_b_* and the reliability factor *γ_x_* were equal to 1.1 and 1.2, respectively.

The Dynamic tests D1, D2, and D3, were performed to evaluate the dependence of the response of the PS-LED on velocity and consisted of five harmonic cycles, each at *d_bd_*, while considering a ±50% variation in frequency with respect to the reference value of 0.5 Hz (Table 1).

Eventually, three tests, S1, S2, and S3, though not requested by the standard [53], were carried out with the aim of assessing the survivability of the damper during repeated sequences of the design earthquake. In these tests, the PS-LED was subjected to three sequences of ten cycles each of sinusoidal displacement at the design seismic displacement *d_bd_*, with a dwell period of about 1 h between two consecutive sequences.

From the experimental load–displacement curves, the effective stiffness *K_eff_* and the equivalent damping ratio *ξ_eff_* of the PS-LED were evaluated at each cycle according to Equations (2) and (3):

(2) $$K_{\mathrm{eff}}=\frac{F_{d}}{d}$$

(3) $$\xi_{\mathrm{eff}}=\frac{2}{\pi }\frac{\mathrm{EDC}}{{4\;d\;F}_{d}}$$

where *EDC* is the energy dissipated per cycle; *F_d_* is the maximum damper force in the cycle (averaged between the positive and negative branches); and *d* is the displacement amplitude.

According to the standard [53], the design properties of hysteretic dampers are determined at the third cycle of test C3. For the prototype under examination, the evaluated values were $K_{\mathrm{eff}}$ = 22.3 kN/mm and *ξ_eff_* = 55%. In this respect, it is worth recalling that the equivalent damping ratio of conventional BRBs generally does not exceed 40%.

The stability of the stiffness and damping properties of the PS-LED prototype in the Cyclic tests were checked in accordance with the standard [53], which prescribes a maximum change of ±10% in *K_eff_* and *ξ_eff_* with respect to the values evaluated at the third cycle performed at the same amplitude (but disregarding the first cycle). The results reported in Table 2 show that the requirement was fulfilled.

**Figure 4:**
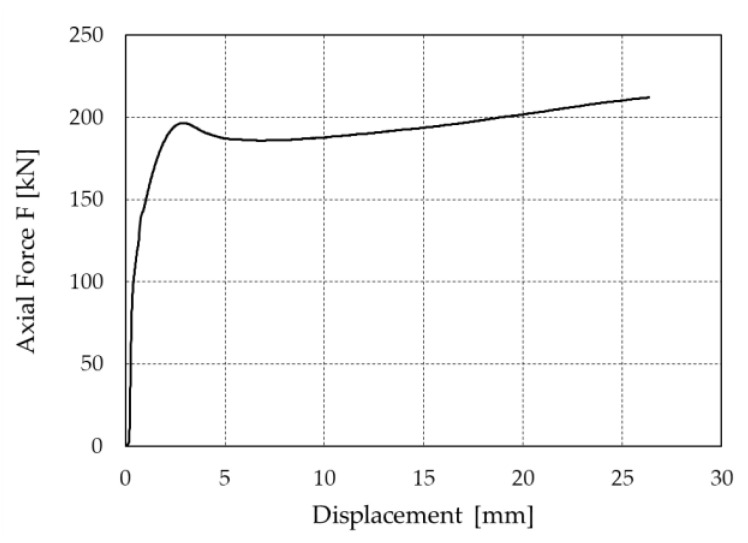
Force–displacement curve of the PS-LED prototype evaluated in the Ramp test.

The force–displacement curve assessed in the Ramp test for the amplified displacement *γ_b_ γ_x_ d_bd_* is shown in Figure 4. The prototype sustains the amplified displacement without any cracking, and after the peak in force in correspondence with the breakaway friction, the force–displacement curve presents a non-decreasing behavior.

The response of the PS-LED was found to exhibit a shallow dependency on velocity, as demonstrated in the Dynamic tests and shown in Table 3. The damper force, and consequently the effective stiffness, has a maximum variation of 4% over a variation in the frequency of +/− 50% with respect to the central frequency of *f* = 0.5 Hz, while the corresponding change in the equivalent damping ratio is about 3%.

**Figure 5:**
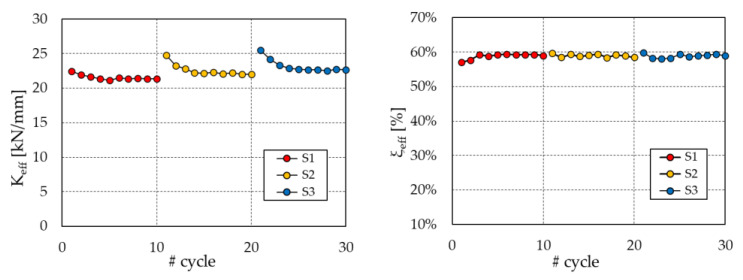
Changes in *K_eff_* and *ξ_eff_* during the tests S1, S2, and S3.

Figure 5 illustrates the variation in the effective stiffness *K_eff_* and the equivalent damping ratio *ξ_eff_* at each cycle of the tests S1, S2, and S3. Disregarding the first cycle of each sequence as recommended in the standard [53], the mechanical properties remained practically unchanged during the tests, with a maximum variation in *K_eff_* and *ξ_eff_*, with respect to the values assessed at the third cycle of S1, of +7.3% and −2.6%, respectively. Eventually, it must be reported that, by adding up all the cycles performed in the experimental program (Table 1), the PS-LED prototype sustained a total of 55 cycles at the design seismic displacement without a deterioration of its properties, demonstrating the absence of low-cycle fatigue effects.

### 2.3. Modeling of the PS-LED in OpenSees

**Figure 6:**
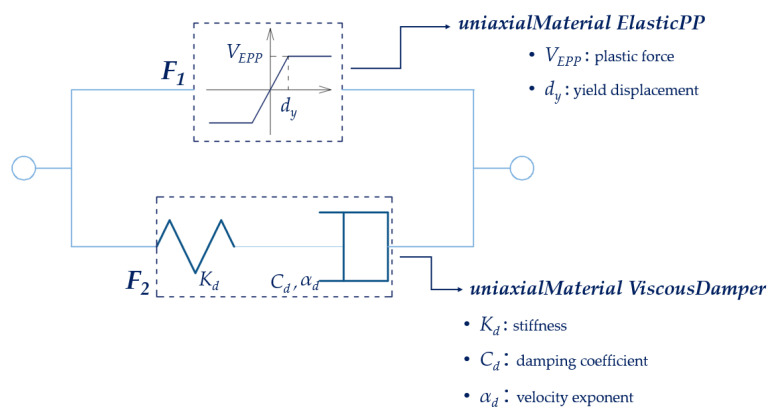
EPPV rheological model in OpenSees.

A rheological model of the PS-LED, named EPPV model [7,29,57], was formulated in the OpenSees framework [54,55] to perform non-linear dynamic analyses. This model is preferred over the velocity-dependent friction models that are already implemented in OpenSees since it allows a direct estimation of the relevant parameters from the Cyclic tests performed for the qualification of the damper according to the standard EN 15129 [53], without requiring additional experimental burden [7]. By referring to the hysteretic cycle produced by a sinusoidal input motion as shown in Figure 3b, the damper shows an almost elastic-perfectly plastic behavior, with “rounded” corners at the motion reversals, where the velocity is close to zero. These variations are due to the shallow dependence of the damper force on the rate of displacement, as confirmed by the Dynamic tests reported in Section 2.2. To describe such behavior, an in-parallel model was formulated (Figure 6), which includes an elastic-perfectly plastic material and a viscous Maxwell material. In particular, the contribution of the elastic-perfectly plastic material is identified with the force *F_1_*, while *F_2_* is the force of the Maxwell material.

The EPPV model was coded in OpenSees [54,55] by using a *zeroLength element* [55] associated with a *Parallel* material, which includes two material objects, the *uniaxialMaterial ElasticPP* [55] and the *uniaxialMaterial ViscousDamper* [55,58]. This model is defined by 5 parameters: the yield displacement $d_{y};$ the plastic force $V_{EPP}$ of the *uniaxialMaterial ElasticPP* material object; the stiffness $K_{d}$; the damping coefficient $C_{d};$ and the velocity exponent $\alpha_{d}$ of the *uniaxialMaterial ViscousDamper* material object. 

By referring again to the hysteretic cycle shown in Figure 3b, the model parameters for the tested damper are determined as follows:

(i) *V_EPP_*, which corresponds to the maximum force *F_1_* of the *uniaxialMaterial ElasticPP*, is defined as a fixed share β of the maximum damper force *F_d_*. Then, *F_2_* is simply (1 − β)·*F_max_*.
(ii) *d_y_* is defined on the initial branch of the force–displacement curve as the displacement at which *V_EPP_* is reached first.
(iii) *K_d_* is set equal to 100·(*V_EPP_*/*d_y_*), in order to concentrate the whole deflection of the Maxwell element in the dashpot.
(iv) *C_d_* is defined as the ratio *F*_2_/(*v_max_*)*^αd^*, where *v_max_* is the maximum velocity in the cycle and *α_d_* is the velocity exponent, which value is determined by minimizing the difference between the areas of the experimental and the analytical loops.

**Figure 7:**
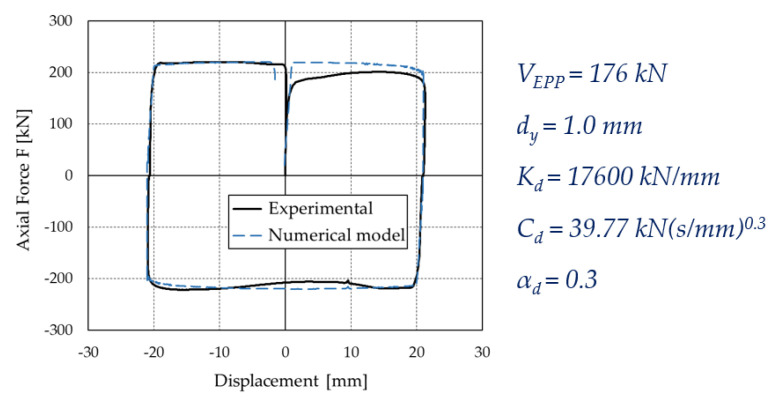
Matching of the numerical model with the experimental curve, and identified parameters of the EPPV model, adapted from [7].

Figure 7 reports the model parameters identified for the investigated prototype of the PS-LED and shows the fair agreement between the experimental curve and the analytical model. The largest discrepancy between the two curves occurs in the first quadrant of the diagram, where the damper does not reach its maximum force due the inertia of the testing machine at the breakaway. The maximum force in tension and compression and the effective stiffness deviate by less than 1% between the model and the experiment, while for the *EDC,* the difference is on the order of 3% if considering the whole cycle, and less than 1% if ignoring the first quadrant.

## 3. Assessment of the PS-LED for the Retrofit of Existing Structures

### 3.1. Description of the Case-Study Buildings

Two existing frame structures taken from the literature [9,59] were assumed as the case studies, namely a residential RC building and a steel office building. These structures were chosen since they had been designed according to outdated codes, and they are characterized by some deficiencies in structural design that can be considered typical in existing structures.

**Figure 8:**
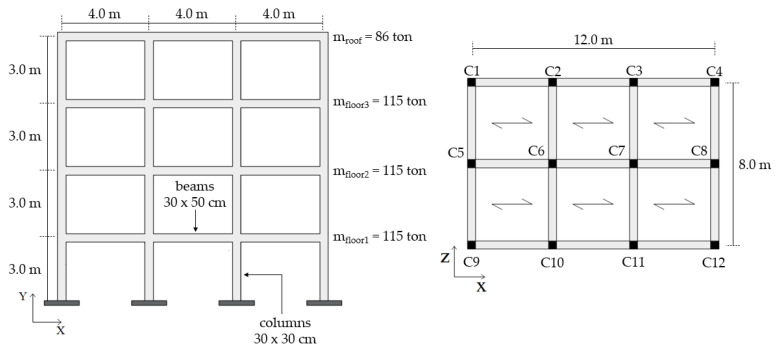
Elevation view and plan with the main dimensions of the RC case-study structure [9].

**Figure 9:**
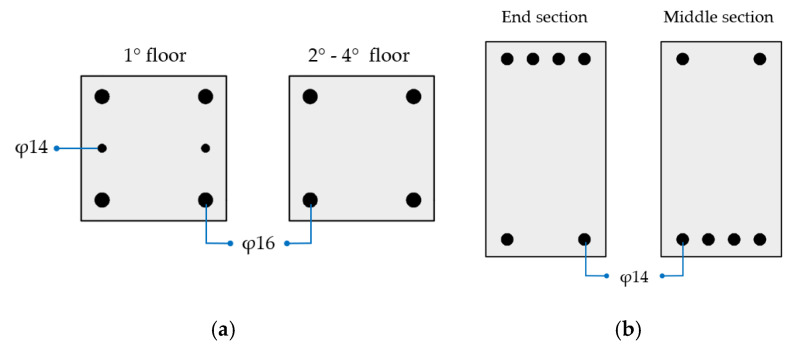
Reinforcement of (**a**) columns and (**b**) beams.

The RC building is a 4-story structure located in the municipality of Potenza (Italy), which corresponds to a medium/high seismic area according to the classification of NTC-18 [56]. The elevation and plan view of the building, with the relevant main dimensions, are illustrated in Figure 8. The column and beam longitudinal reinforcements are shown in Figure 9; concerning the transversal reinforcement, the columns have φ6 stirrups spaced at 15 cm, whereas the beams have φ6 stirrups spaced at 15 cm at the end sections and at 20 cm elsewhere. The compressive strength of concrete is 20 MPa, and the yield strength of steel is 375 MPa; additional information can be found in reference [9]. The building was designed in the 1980s by considering vertical loads only and disregarding the effect of earthquake. Only the collapse of the case-study structure in bending is considered, while other possible failure mechanisms, e.g., shear failure of beams, columns or beam–column joints, bond slip, and low-cycle fatigue, which are rather common for old-code buildings [31], are not considered in the present work.

**Figure 10:**
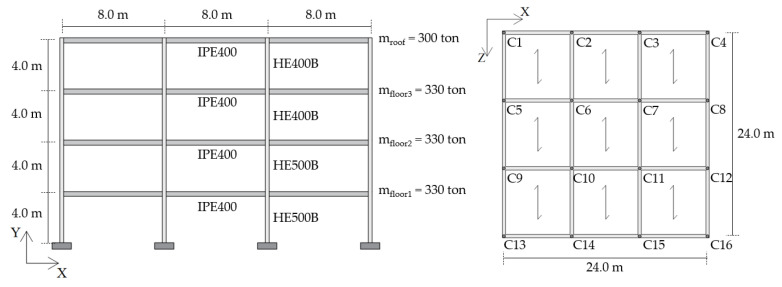
Elevation view and plan with the main dimensions of the steel case-study structure [59,60].

The steel MRF building [59,60,61] is shown in Figure 10, where the section and the length of the structural elements are reported. The peculiarity of this building consists of presenting different stiffnesses and strength capacities in the two main horizontal directions, due to the preferred orientation of the column sections, with their strong direction aligned to the Z-axis. The beams and columns are made of S355 steel.

### 3.2. Modeling of the Case-Study Frames in OpenSees

Full 3D numerical models were formulated in the OpenSees framework [54,55]. Both buildings had fixed supports at the ground floor to simulate rigid foundations, and the floor slabs were modeled as rigid diaphragms. Seismic masses at each floor, evaluated in compliance with the Italian code NTC-18 [56], were concentrated at the master nodes. Dead and live loads, as provided in the relevant references [9,59], were uniformly applied to the beams according to the warping shown in Figure 8 and Figure 10. Following [62,63], the beams and columns were modeled using the *forceBeamColumn* element object [64], which is composed of two external sub-elements of length *L_pl_* corresponding to the end regions where inelastic behavior can be triggered, and an internal sub-element characterized by a linear elastic behavior. A different *L_pl_* length was assumed for either case-study structure, namely, Equation (4) for the RC building and Equation (5) for the steel MRF.

(4) $$L_{pl}=\frac{z}{30}+0.2h+0.11\left(\frac{d_{b}f_{y}}{\sqrt{f_{c}}}\right)$$

Equation (4) is equivalent to the provision of the Eurocode 8 [65], where *z* is the shear span of the structural element, *h* is the depth of the section, *d_b_* is the diameter of the longitudinal rebar, *f_y_* is the yield strength of steel, and *f_c_* is the compressive strength of concrete. According to the norm [65], Equation (4) is valid when a well-detailed confinement model of concrete is assumed [62]. For this reason, for the concrete model, the uniaxial material *Concrete04* [55], which is based on the model proposed by Popovics [66], was implemented. Each steel bar corresponds to a single fiber, which is associated with the uniaxial Giuffre–Menegotto–Pinto constitutive law [67], corresponding to the *Steel02* material with isotropic strain hardening [68] coded in the OpenSees libraries. A strain hardening ratio *b* = 0.01 was assumed, and the parameters that control the transition from the elastic to the plastic branch were set as *R0* = 18, *CR1* = 0.925, and *CR2* = 0.15 [55]. In order to account for concrete cracking in the internal elastic sub-element, an effective area moment of inertia $I_{eq}$ defined as 50% of the gross area moment of inertia $I_{g}$ was introduced, according to the provisions of the NTC-18 [56].

For the steel MRF, Equation (5) was used [69,70], where *L_v_* is the shear length of the steel member:

(5) $$L_{pl}=0.22\;L_{v}$$

Again, the *Steel02* material model with isotropic strain hardening [68] was implemented, where the yield strength $f_{y}$, the modulus of elasticity $E_{s},$ and the strain hardening ratio b were assigned to be equal to 355 MPa, 210,000 MPa, and 0.01, respectively.

The design properties of the materials were evaluated without considering the confidence factors [56,65]. P-Delta effects were considered in the analyses, and while the infill panels were not modeled, their contribution was taken into account as additional energy dissipation; indeed, equivalent viscous damping ratios of 5% for the RC frame (as in references [7,71,72,73]) and of 3% for the steel MRF (as in references [61,74,75]) were assumed.

Finally, an “axial buffer”, modeled as a *zeroLength* element object [55] with zero stiffness in the axial direction and high stiffnesses in shear and bending, was introduced in the RC frame model between one end of each beam and the adjacent node, in order to eliminate the axial force fictitiously generated by the interaction between the fiber sections of the beam elements and the rigid diaphragm [76].

### 3.3. Design of Seismic Retrofit with Chevron Braces Equipped with the PS-LED

The seismic upgrade of the two structures was designed by referring to the seismic loads provided by the NTC-18 [56] for life-safety limit state (SLV). In particular, the RC structure was upgraded considering the seismic hazard associated with the municipality of Potenza (latitude 40.65°, longitude 15.81°), with PGA = 2.45 m/s^2^, soil type B, and topographic factor T_1_ for a building with a functional class *c_u_* = II and a nominal life *V_n_* = 50; the retrofit of the steel MRF considered the seismic hazard for the municipality of Lamezia Terme (latitude 38.57°, longitude 16.18°), with PGA = 4.47 m/s^2^, soil type C, and topographic factor T_1_ for a building with a functional class *c_u_* = II and a nominal life *V_n_* = 100.

The dampers were sized by applying the DDBD retrofit procedure developed by the authors of this work [61,71,72,73]. According to this procedure, both the main frame and the dissipative braces are replaced by equivalent single-degree-of-freedom (SDOF) models, each one characterized by a secant stiffness and an equivalent viscous damping; the parameters of the equivalent SDOF damped brace are, hence, defined in relation to a “performance point” with a target displacement $d_{p}$, which is assigned on the basis of the allowable damage of the main frame. In the last step, the strength and stiffness of the damped braces are distributed at each floor according to a proportionality criterion, in order to constrain the retrofitted frame to follow the first mode displacement of the bare frame. Further details can be found in references [2,29,61,71,72,73].

**Figure 11:**
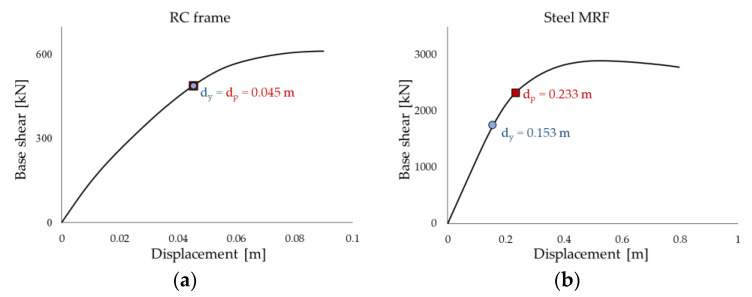
Capacity curves in the X-direction and limit values of displacements for the two case-study structures: (**a**) RC building, and (**b**) steel MRF building.

Different performance requirements were assumed for the two buildings. For the RC structure, the retrofit target was to avoid inelastic deformation of the structure under the effect of the basic design earthquake. In particular, the target displacement $d_{p}$ was defined, in agreement with reference [77], as the ending point of the elastic part of the capacity curve, as shown in Figure 11a. This performance level corresponds to the Immediate Occupancy performance level, which guarantees that the structure is immediately accessible after a main earthquake, since the strength and the stiffness of the structural elements are not compromised. On the contrary, in the case of the steel MRF, a controlled inelastic deformation of the main frame was permitted, with activation of plastic hinges and limited reparable damage, corresponding to a ductility factor of µ_F_ = 1.5 in accordance with the figure assumed in previous studies [59], as shown in Figure 11b.

Figure 11 shows the capacity curves of the two case-study structures in the X-direction; the blue dots on the curves identify the ending point of the elastic part, while the red dots highlight the assumed performance point. For the sake of conciseness, the results in the Z-direction are not reported, but the target displacement is 0.045 m for the RC structure and 0.265 m for the steel MRF, respectively. The seismic analyses of the bare frames (not reported in this paper for brevity) highlighted that, due to the preferred orientation of the column sections with their strong axis aligned in the Z-direction, the steel MRF needed to be upgraded in the X-direction only; interested readers can refer to [61] for more details. On the other hand, the RC frame needed to be strengthened in both directions.

**Figure 12:**
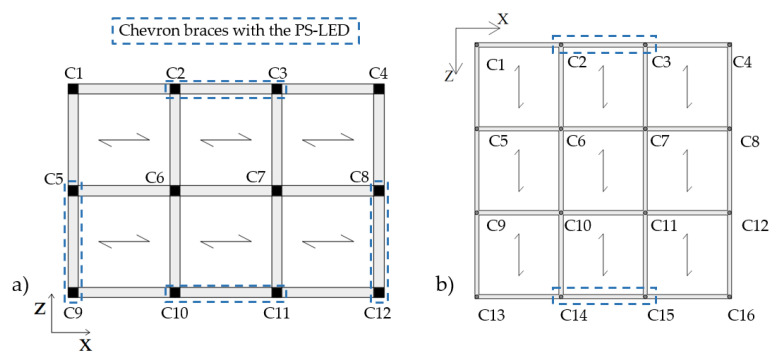
Plan layout of the Chevron braces equipped with the PS-LED: (**a**) RC structure, and (**b**) steel MRF.

**Figure 13:**
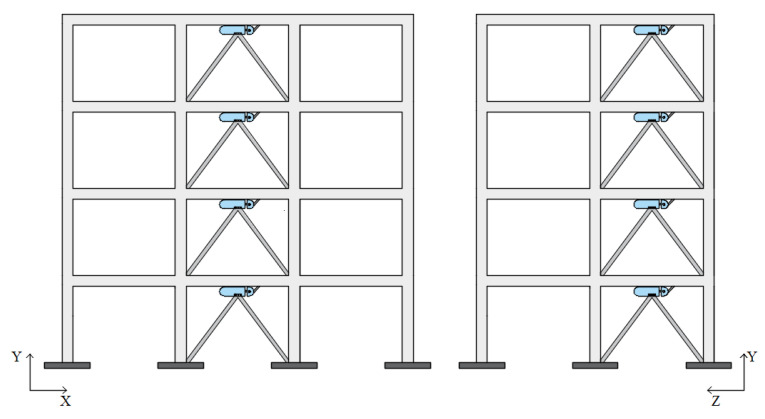
Elevation layout of Chevron braces equipped with the PS-LED in the RC case-study structure.

The two structures were retrofitted by means of Chevron (or reversed-V) steel braces with the PS-LED placed at the intersection of the two rods of the brace and connected to the midsection of the beam of the upper floor. Figure 12 and Figure 13 show the plan layout of the damped braces in both case-study buildings and the elevation layout of the braces in the RC structure, respectively.

**Figure 14:**
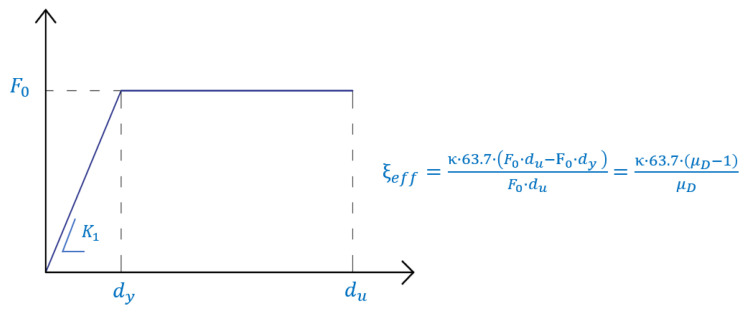
Force–deflection diagram of a single brace equipped with the PS-LED.

For design, the PS-LED were modeled as elastic-perfectly plastic devices, as shown in Figure 14. The design properties of the model are the yield force *F*_0_, the elastic stiffness *K*_1_, and the ductility factor μ_D_ = *d*_u_/*d*_y_. All other parameters of the ideal curve can be derived from these properties.

The steel braces were assigned to have a stiffness twice the PS-LED stiffness, as suggested, e.g., in [9], in order to guarantee that the largest part of the deformation of the story is concentrated in the damper, which enhances the amount of energy dissipation [78,79].

The properties of the dissipative braces are distributed at each story according to a proportionality criterion [80], which ensures that the first modal shape of the bare frame remains unvaried after the upgrade. This choice, which is justified by the vertical regularity of the buildings, avoids drastic changes to the internal action distribution in the frame, at least in the range of the elastic behavior. Moreover, the chosen distribution of strength of the dissipative braces aims at achieving simultaneous yielding of the devices at all the stories and, thus, a global ductility of the bracing system coinciding with the ductility of the single brace [81]. Table 4 and Table 5 report the values of *F*_0_ and *K*_1_ of the dampers at each floor of the RC structure and of the steel MRF, respectively, as evaluated from the design procedure assuming μ_D_ = 20, which corresponds to an equivalent viscous damping ratio *ξ_eff_* = 55% (Figure 14).

It is worth mentioning that Table 4 and Table 5 list the design properties of the PS-LED units obtained from the sizing procedure. In a real application, at this step, the designer would choose from the portfolio of the manufacturer the devices that better match the design values, while also taking into consideration other practical issues, such as the need to use few sizes in order to reduce manufacturing costs [7]. However, for the scope of the present paper, the verification of the upgraded buildings was performed by referring to the design values listed in the above tables.

### 3.4. Seismic Performance and Evaluation of Retrofit Effectiveness

The effectiveness of the PS-LED for the seismic retrofit was investigated by means of bidirectional NLDAs, which were performed in compliance with the NTC-18 [56], considering seven pairs of artificial accelerograms generated by the computer program SIMQKE [82]. The accelerograms had a duration of the pseudo-stationary part of 10 s and a total duration of 25 s, and the mean 5% damping elastic spectrum calculated from all time histories was compatible with the target 5% damping elastic response spectrum defined by the code [56] in the range of periods between 0.15 and 2 s. Artificial accelerograms with a smooth spectrum were chosen as they allow a more accurate control of the frame response than real accelerograms, making the interpretation simpler and more focused on the specific aspects that were investigated in the analyses [7,71]. In the OpenSees models [54,55], the PS-LED was formulated as a concentrated zero-length element (*zeroLength* element object in the OpenSees framework [55]) with the associated EPPV rheological model presented in Section 2, while the steel braces were represented as truss elements with a perfectly elastic behavior (*uniaxialMaterial Elastic* [55]).

**Figure 15:**
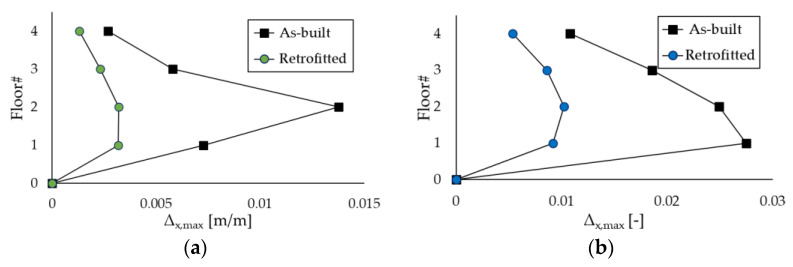
Maximum inter-story drift ratio Δ in the X-direction obtained by NLDAs: (**a**) RC structure, and (**b**) steel MRF.

**Figure 16:**
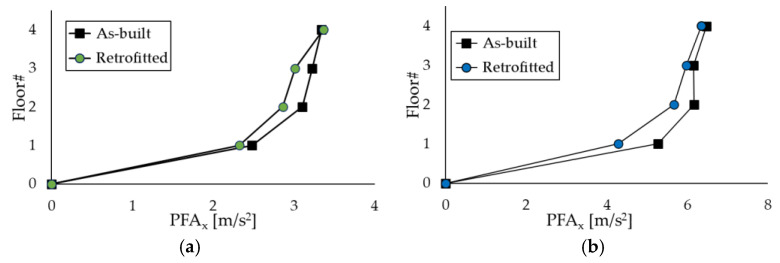
Peak floor acceleration *PFA* in the X-direction obtained by NLDAs: (**a**) RC structure, and (**b**) steel MRF.

The benefits of introducing Chevron braces equipped with the PS-LED in the case-study structures are illustrated in Figure 15 and Figure 16, which show the maximum inter-story drift ratio Δ and the peak floor acceleration (*PFA*) at each story calculated by averaging the maxima of the results obtained for each pair of accelerograms. For the sake of brevity, the results reported in the Figures refer to the X-direction only, but the findings in the Z-direction for the RC frame are included in the discussion as well.

**Figure 17:**
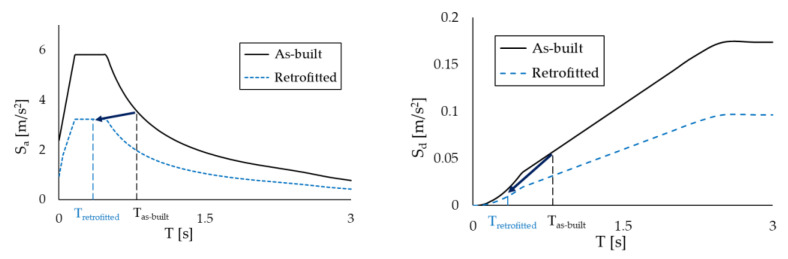
Acceleration and displacement spectra for the RC frame in the as-built and retrofitted configurations, showing the combined effect of structural stiffening introduced by the brace and damping of the PS-LED.

**Figure 18:**
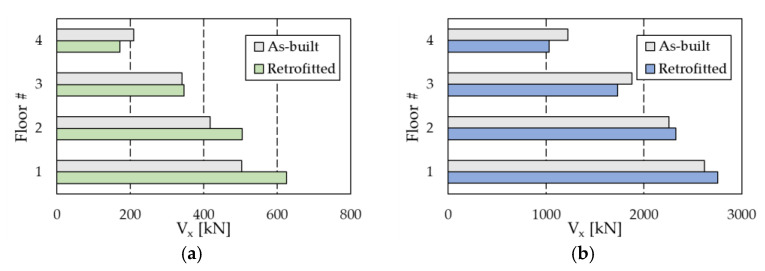
Maximum shear force *V* in the X-direction: (**a**) RC structure, and (**b**) steel MRF.

Figure 15 highlights the substantial reduction in Δ passing from the as-built frames to the retrofitted configurations, which is combined with a small decrement in *PFA*, due to the damping provided by the PS-LED (Figure 16). The upgraded structures show drift ratios that are more than 50% lower than those of the as-built frames, with a maximum decrease of −76.7% at the second floor of the RC frame and −66.6% at the first floor of the steel MRF, respectively. It is noted that with conventional dampers, the structural stiffening introduced by dissipative braces is normally accompanied by an increase in floor acceleration [23]. In contrast, with the PS-LED, due to the combined effects of stiffening provided by the braces, which decreases the vibration period by reducing displacements but increasing accelerations, and damping introduced by the PS-LED, which lowers the response spectra, it is possible to control the deformation of the building and the structural acceleration at the same time (Figure 17). The benefit of energy dissipation introduced by the PS-LED is also evident when looking at Figure 18, which shows the maximum shear force *V* at each floor. For both structures under the basic design earthquake, the retrofitted configurations exhibit a small increase in the base shear, which is on the order of 24.5% for the RC frame and of 5% only for the steel MRF. Therefore, the use of the PS-LED may reduce the need for invasive and expensive strengthening of the foundations, which is usually required when hysteretic steel dampers are used.

It is interesting to note in Figure 18 that for both buildings at the upper floors, the maximum shear force of the retrofitted frame is smaller than that of the as-built structure, but the opposite occurs at the lower floors. This behavior can be explained by noticing in Table 4 and Table 5 that the yield force of the PS-LED damper increases toward the lower floors. Specifically, at the fourth floor, the force of the PS-LED damper is about one third of the force of the device at the first floor. However, at each story, regardless of the damper force, the overall damping introduced in the building by the damped braced systems produces a consistent decrease in floor accelerations. For this reason, at the upper floors (third and fourth floors of the steel MRF, and fourth floor of the RC frame), the shear force of the retrofitted building is smaller than that of the as-built structure. On the contrary, at the lower floors, the force of the PS-LED overcomes the reduction in the inertia force due to the lower acceleration, resulting in a moderate increase in the total shear.

**Figure 19:**
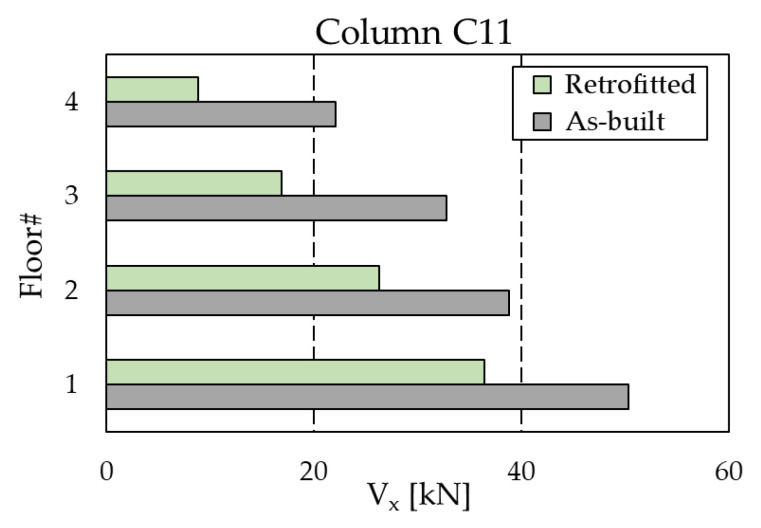
Maximum shear forces in the most stressed column (position C11 in Figure 12a) of the RC structure.

**Figure 20:**
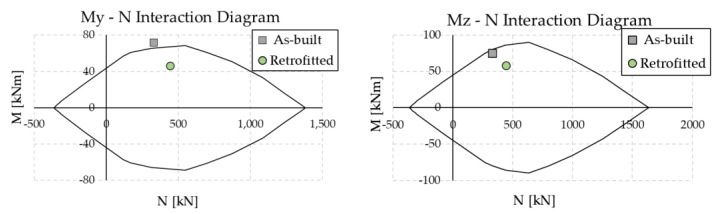
Check of column C11 (Figure 12a) at the ground floor of the RC structure in the M–N interaction diagram.

Figure 19 compares the maximum shear force in the most stressed column (corresponding to position C11 according to Figure 12a) at every floor of the RC frame. The introduction of the dissipation braces produces a substantial decrease in the shear force at each story; for example, at the ground floor, where the largest value is attained, the force passes from 50.3 kN in the as-built configuration to 36.4 kN in the upgraded configuration. This effect is ascribed to the Chevron braces, which create an alternative channel for the horizontal forces, reducing the amount of shear force transmitted through the columns. However, the vertical component of the force through the inclined braces is transferred to the columns of the lower floors, which are therefore subjected to a higher axial force. The columns at the ground floor of the RC structure were checked in the moment–axial load (M–N) interaction diagram. Figure 20 reports the results for the element at position C11. As shown in the panels, the check is not verified in the as-built configuration, and the column is expected to collapse in flexure. In contrast, the introduction of the dissipative braces causes both a reduction in the bending moment (related to the reduction in shear force) and an increase in the axial force, which globally provide a fulfillment of the resistance check.

**Figure 21:**
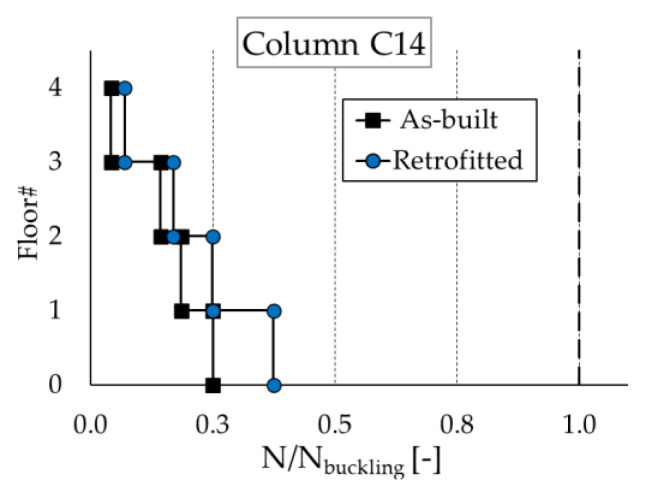
Buckling check of column C14 (Figure 12b) of the steel MRF.

On the other hand, a typical issue for steel frames is represented by the buckling of slender columns. The relevant check was, therefore, performed in order to evaluate the effect of an increased compressive force on the columns of the retrofitted steel structure. The results for the most stressed column (corresponding to position C14 according to Figure 12b) are shown in Figure 21, where the maximum axial force of the column at each floor is compared to the relevant buckling load (*N_buckling_*) calculated according to the NTC-18 [56]. Despite a non-negligible increase in the axial force (at the ground level, the axial load rises from 1035.3 kN to 1549.0 kN), the check is verified.

In order to highlight the contribution of energy dissipation provided by the PS-LED, the energy time histories of the frame and of the dampers are evaluated. In particular, the absolute input energy [83] is determined at each floor according to the expression of Equation (6):

(6) $$E_{Ij}=\int_{0}^{t}m_{j}{ϋ}_{tj}d\upsilon_{g}$$

where the index “*j*” refers to the *j*th story, and the relevant parameters are *m_j_*, the mass associated with the *j*th story; *ϋ_tj_*, the absolute acceleration of the *j*th story; and *υ_g_*, the ground displacement.

**Figure 22:**
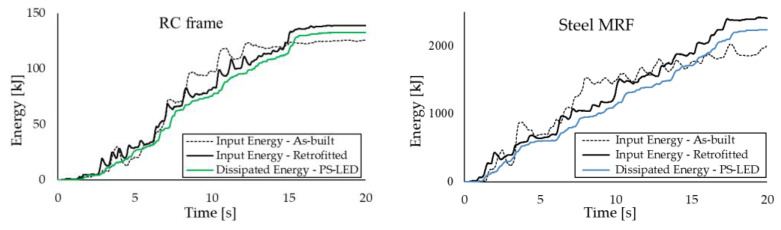
Comparison of energy time histories of the buildings and the total energy dissipated by the PS-LED dampers calculated for the most demanding input accelerogram.

**Figure 23:**
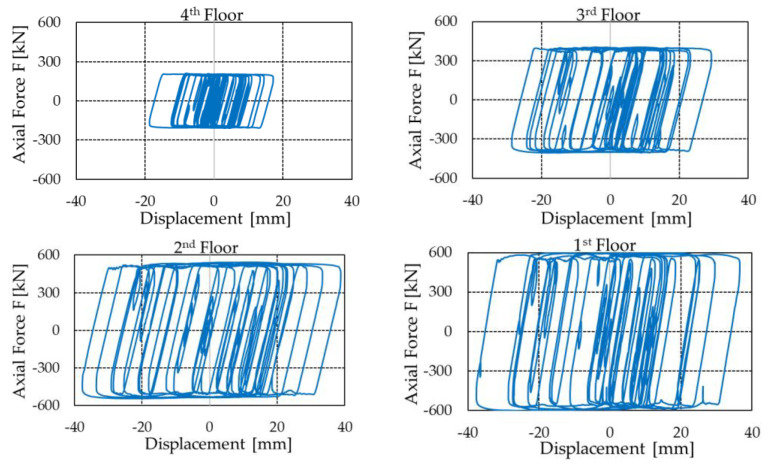
Force–displacement cycles of the PS-LED dampers at each floor of the steel MRF calculated for the most demanding input accelerogram.

In Figure 22, the absolute input energy introduced by the most demanding input accelerogram is compared with the energy dissipated by the PS-LED dampers installed in the perimetral frames in the X-direction of the upgraded structures. The energy dissipated by the PS-LED is calculated as the sum of the areas enclosed in the hysteretic cycles. As an example, the force–displacement cyclic behavior of the dampers installed at every floor of the steel MRF is reported in Figure 23. These loops highlight the excellent agreement between the actual strength of the dampers and the design values reported in Table 5, confirming the effectiveness of the proposed EPPV model. As shown in Figure 22, in the case of the RC structure, the dampers dissipate about 95% of the total input energy, while in the case of the steel MRF, the dissipated energy is about 92% of the input energy. By recalling that an equivalent viscous damping ratio of 5% was assumed for the RC frame, and a ratio of 3% was assumed for the steel MRF, these results are in fair agreement with the target of the retrofit design, which aimed at guaranteeing an essentially elastic behavior of the RC frame and permitting limited inelastic deformations of the steel frame. It is also worth noting that, for both structures, the input energies calculated in the as-built and retrofitted configurations are quite close to each other. However, in the as-built configuration, when disregarding the small contribution of the structural viscous damping, the input energy is totally dissipated through the inelastic deformation of the structural members, which therefore undergo severe damage.

## 4. Conclusions

The present work deals with a novel damper, named the PS-LED, which dissipates the input seismic energy through the friction force triggered between a steel shaft and a lead core prestressed within a rigid steel chamber. The friction force is adjusted by controlling the prestress of the core, allowing the achievement of high forces with small dimensions, which reduces the architectural invasiveness of the device. The damper was experimentally investigated, and a numerical model was coded in the OpenSees framework [54,55]. To assess the viability of the PS-LED for the seismic upgrade of existing buildings, a RC frame and a steel frame were considered as the case studies. Steel braces equipped with the PS-LED were designed to obtain a retrofitted structure that is able to withstand, at a specified performance level, the seismic demand associated with the life-safety limit state. Successively, the performance of the retrofitted buildings was evaluated by performing NLDAs under a set of artificial ground motion records. The main conclusions of the study are as follows:

(i) The prototype of the PS-LED exhibits a stable response during repeated cycles performed at the design displacement, fulfilling the qualification requirements of the European standard [53] and providing an equivalent damping ratio on the order of 55%. Differently from current hysteretic dampers, such as buckling-restrained braces, the novel damper has no mechanical parts subjected to cyclic strain above their yield limit; therefore, the risk of low-cycle fatigue is avoided, and the device is expected to be able to withstand an unlimited number of cycles without deterioration or failure.
(ii) A rheological model of the PS-LED was formulated in the OpenSees software program, consisting of an in-parallel arrangement of two material objects, namely an elastic-perfectly material (*uniaxialMaterial ElasticPP*) and a Maxwell model (*uniaxialMaterial Viscous Damper*). The model is able to reproduce the fundamental behavior of the damper, including the light dependency on velocity highlighted by the tests, and provides accurate predictions of maximum force, effective stiffness, and energy dissipation.
(iii) In the application examples, the PS-LED dampers are shown to dissipate the largest part of the input energy introduced in the structure by the design earthquake, guaranteeing the protection of the main frame at the level specified by the design strategy (either elastic behavior or controlled inelastic deformation).
(iv) A consequence of the excellent dissipative behavior of the PS-LED is the ability, in the examined case studies, to control the shear forces in the columns and foundations of the retrofitted frame; therefore, additional strengthening of the main structure may be not necessary or may be limited, resulting in a potential reduction in the total cost of the retrofit intervention.

Although the experimental investigation was restricted to a single size of the device, this study highlights the potential advantages of the PS-LED over current steel dampers for improving the survivability of structures to repeated ground motions, such as during the main shock–aftershock sequences, as well as reducing the need for local strengthening of the main frame. Future developments of the present work will concern the testing of prototypes with different yield forces and displacement capacities in order to evaluate the effect of the dimensions of the device on mechanical behavior, and to investigate experimentally the relationship between the prestress applied to the lead core and the strength of the PS-LED, with the aim of deriving design charts that can be used by manufacturers for the mechanical sizing of the device. Additionally, the performance of buildings reinforced with the PS-LED will be investigated while considering different seismic scenarios, such as those corresponding to service limit states and ultimate limit states.

## Figures and Tables

**Table 1:** Testing protocol according to the EN 15129 [53].

Test		*d* [mm]	*f* [Hz]	No. of Cycles [-]
Cyclic	C1	5	0.5	5
	C2	10	0.5	5
	C3	20	0.5	10
Ramp	R	26.4	0.001	1
Dynamic	D1	20	0.25	5
	D2	20	0.50	5
	D3	20	0.75	5
	S1	20	0.5	10
Survivability	S2	20	0.5	10
	S3	20	0.5	10

*d*: cycle amplitude; *f*: test frequency.

**Table 2:** Stability requirements [53] and results of the tests on the PS-LED prototype.

Requirement	C1 *d* = 5 mm	C2 *d* = 10 mm	C3 *d* = 20 mm
$\frac{\left|K_{\mathrm{eff},i}-K_{\mathrm{eff},3}\right|}{K_{\mathrm{eff},3}}\;\leq \;0.10$	+1.8%	−5.2%	−9.3%
$\frac{\left|\xi_{\mathrm{eff},i}-{\;\xi }_{\mathrm{eff}3}\right|}{\xi_{\mathrm{eff},3}}\;\leq \;0.10$	−1.3%	−3.6%	−2.2%

*K_eff,i_* and *ξ_eff,i_* are the effective stiffness and the equivalent damping ratio evaluated at the i-th cycle (*i ≥* 2), respectively. *K_eff,_*_3_ and *ξ_eff,_*_3_ are the effective stiffness and the equivalent damping ratio evaluated at the third cycle, respectively.

**Table 3:** Effect of velocity on the properties of the PS-LED prototype.

Requirement	D1 *f* = 0.25 Hz	D2 *f* = 0.50 Hz	D3 *f* = 0.75 Hz
$K_{\mathrm{eff},3}$ [kN/mm]	21.8	22.3	23.2
$\xi_{\mathrm{eff},3}$ [%]	53.6	55.3	55.1

**Table 4:** Elastic stiffness $K_{1}$ and yield force $F_{0}$ of the PS-LED dampers installed at each story of the RC structure.

Story	X-Direction		Z-Direction	
	*K*_1_ [kN/mm]	*F*_0_ [kN]	*K*_1_ [kN/mm]	*F*_0_ [kN]
4th	499.6	44.0	499.2	44.5
3rd	529.6	96.0	533.6	97.0
2nd	535.7	133.9	540.9	135.2
1st	644.0	152.4	656.8	153.7

**Table 5:** Elastic stiffness $K_{1}$ and yield force $F_{0}$ of the PS-LED dampers installed at each story of the steel MRF.

Story	X-Direction	
	*K*_1_ [kN/mm]	*F*_0_ [kN]
4th	358.6	207.9
3rd	378.0	400.4
2nd	418.4	531.6
1st	602.3	588.8

## Data Availability

The data presented in this study are available from the corresponding author upon request. The data are not publicly available as they are generated within a research project funded by the Italian Department of Civil Protection.
